# Supplementary material for: Identification and Quantification of Selected Benzoxazinoids and Phenolics in Germinated Spelt (Triticum spelta)
Source: Foods. 2023 Apr 24;12(9):1769. doi: 10.3390/foods12091769 (PMC10178788; doi:10.3390/foods12091769)
Supplement: Supplementary file 1 [file foods-12-01769-s001.zip › foods-2348595-supplementary.pdf]

Supplementary Table S1. Characteristics of the phenolic compounds in germinated spelt extracts as analysed by LC-MS

	**Rt (min)**	**ESI-Precursor ion**	**Fragment ion**	**Standard LOQ ng/column**	**LOD ng/column**	**Sample LOQ ng/column**	**LOD ng/column**	**Recovery (%)**
Schaftoside	10.82	563.16	353.06	0.27	0.08	0.48	0.17	95.66
Isoschaftoside	11.26	563.16	353.06	0.22	0.07	0.44	0.14	93.35
BOA	9.18	133.93	90.93	0.09	0.03	0.21	0.07	94.28
MBOA	11.75	163.95	148.92	0.17	0.05	0.39	0.14	95.34
trans-ferulic acid	11.08	192.95	133.94	0.11	0.04	0.19	0.06	96.78
trans-p-Coumaric acid	9.63	162.90	119.47	0,53	0,16	0,78	0,27	93,77

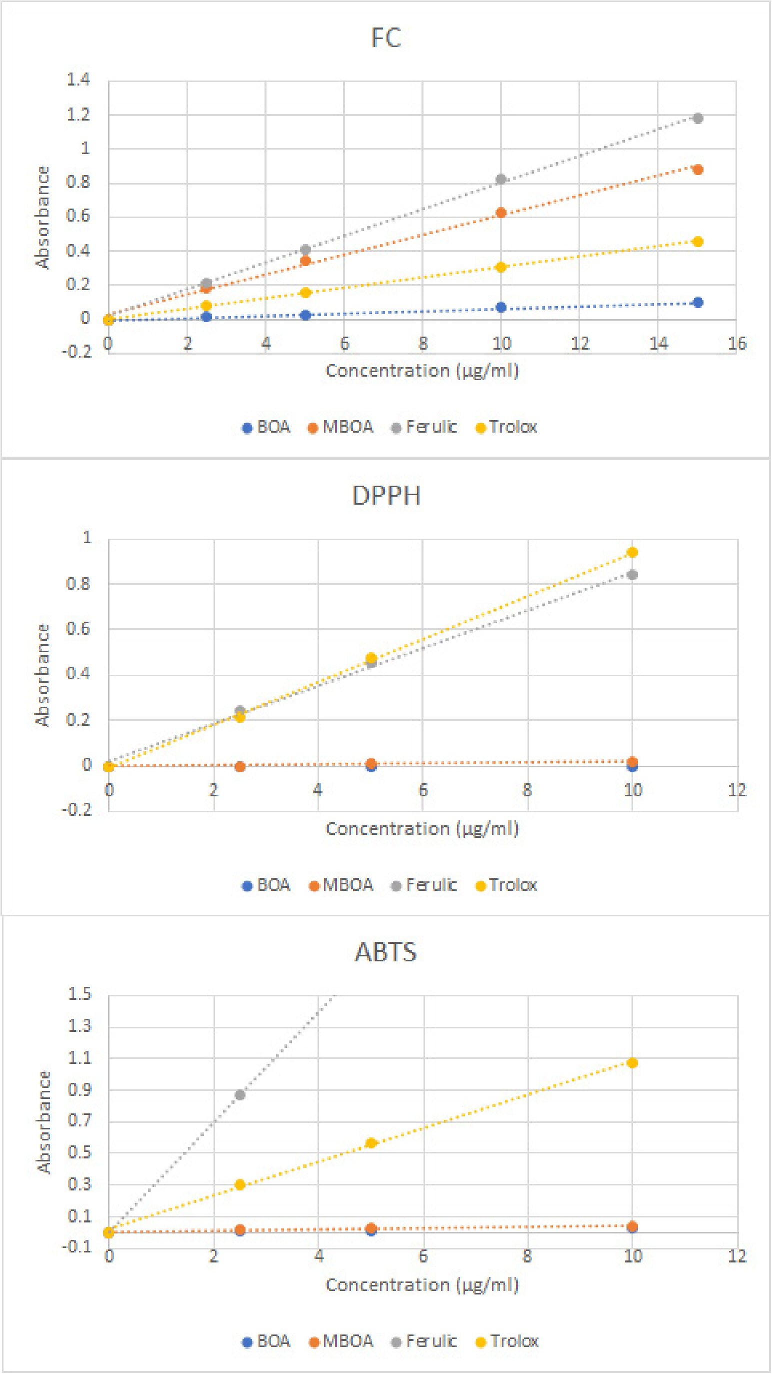

Supplementary Figure S1. Specific reactivities of selected compounds toward FC, DPPH and ABTS reagents

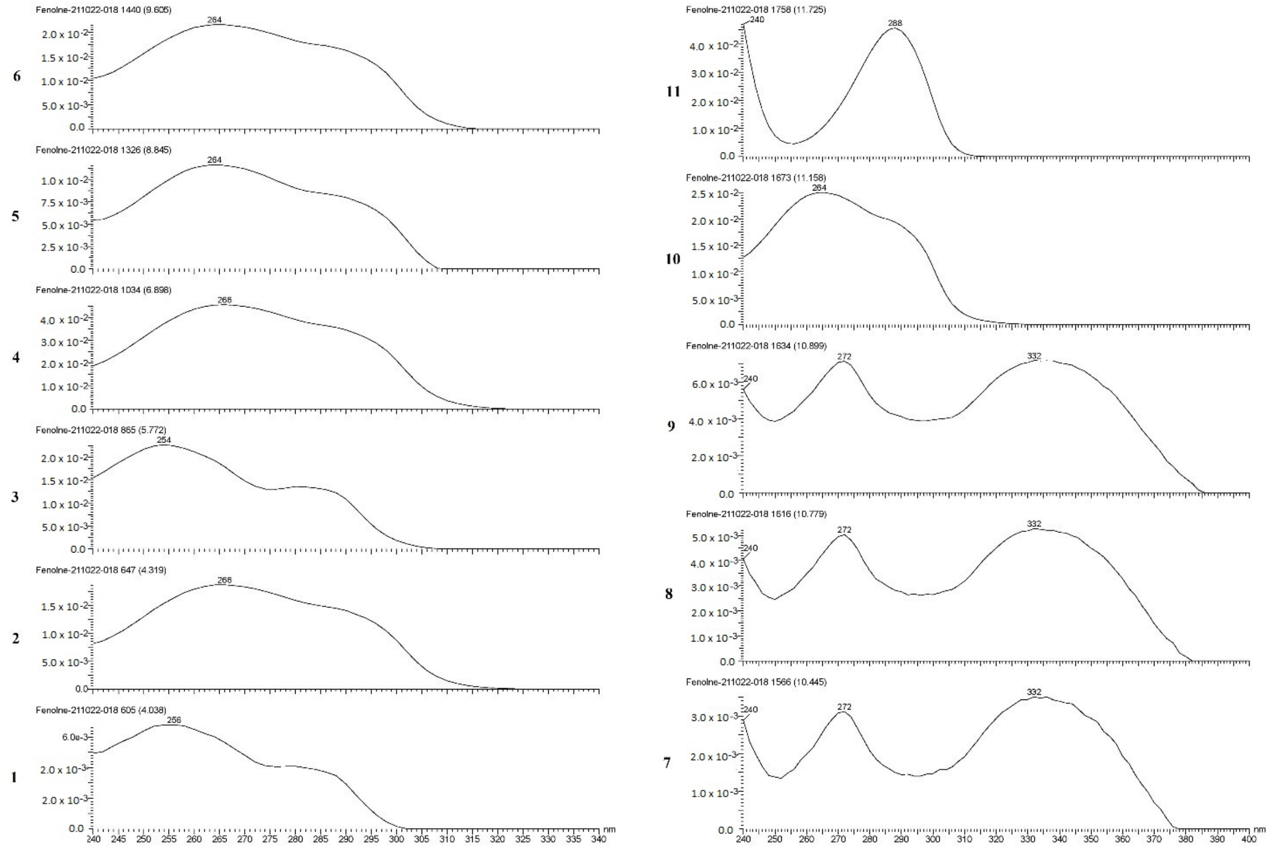

Supplementary Figure S2. UV spectrum of selected compounds in germinated spelt extracts. **1**- Unidentified, (m/z 534.3); **2**- Unidentified, (m/z 534.3); **3**- Unidentified, (m/z 134.2); **4**-Unidentified, (m/z 418.2); **5**-HBOA; **6**-Unidentified, (m/z 594.5); **7**- Shaftoside structural isomer 1; **8**- Shaftoside; **9**- Shaftoside structural isomer 2; **10**-Unidentified (m/z 432.4); **11**- MBOA.

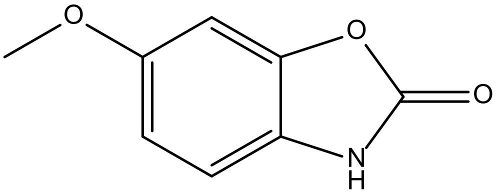

Supplementary Figure S3. Structure of 6-Methoxy-2-benzoxazolinone (MBOA)

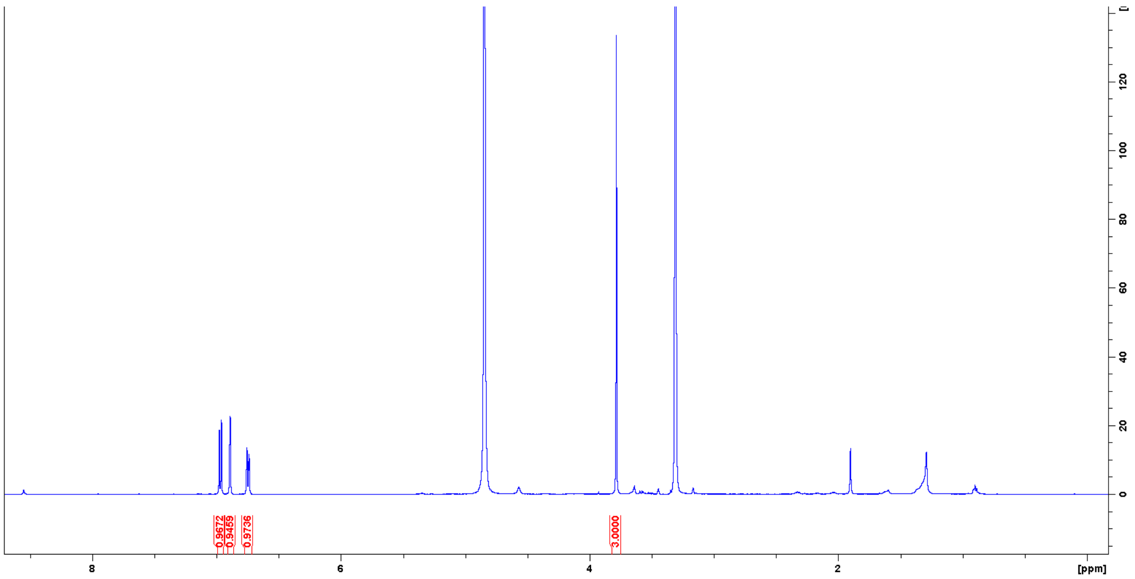

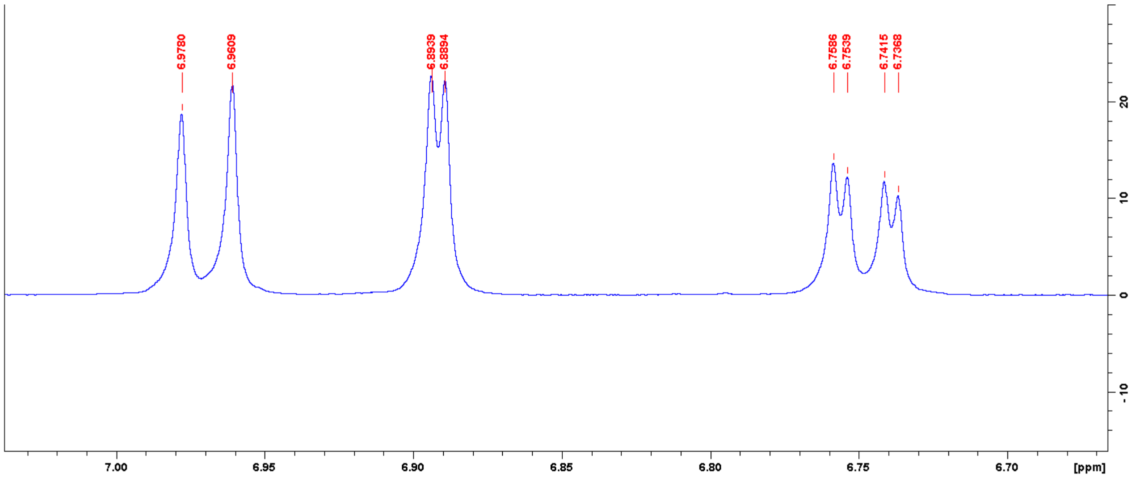

Supplementary Figure S4. ^{1}H NMR spectrum of MBOA

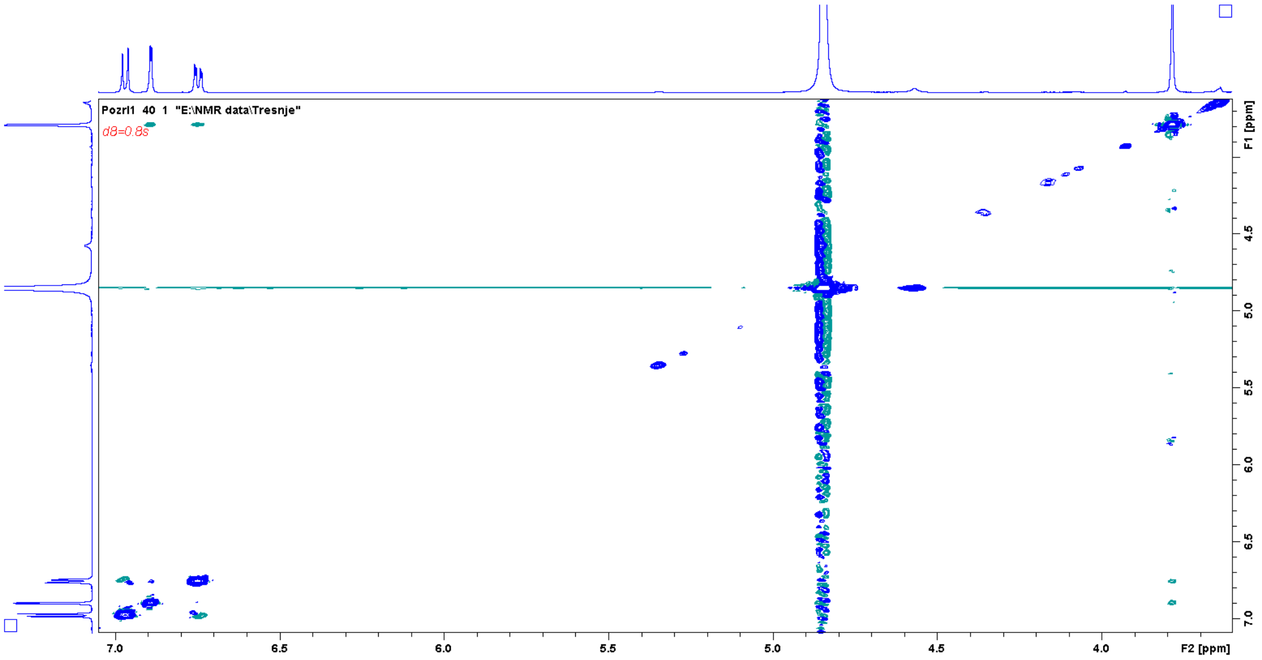

Supplementary Figure S5. NOESY spectrum of MBOA

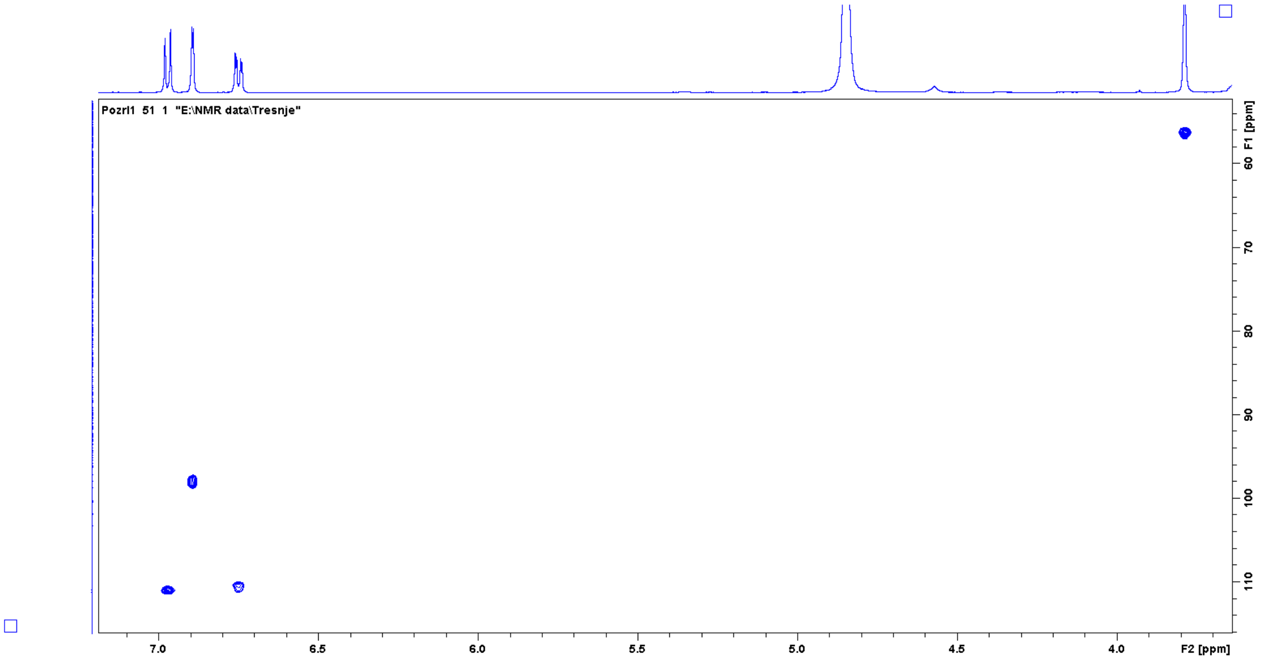

Supplementary Figure S6. HSQC spectrum of MBOA
